# Supplementary material for: Aberrant methylation underlies insulin gene expression in human insulinoma
Source: Nat Commun. 2020 Oct 15;11:5210. doi: 10.1038/s41467-020-18839-1 (PMC7566641; doi:10.1038/s41467-020-18839-1)
Supplement: Supplementary file 1 — Supplementary Information [file 41467_2020_18839_MOESM1_ESM.pdf]

Supplementary Information For:

Aberrant Methylation Underlies Insulin Gene Expression in Human Insulinoma

[1,¶]Esra Karakose PhD, [2,¶]Huan Wang PhD, [1]William Inabnet MD, [3]Rajesh V. Thakker MD, [4]Steven Libutti MD, [1]Gustavo Fernandez-Ranvier MD, [1]Hyunsuk Suh MD, [3]Mark Stevenson PhD, [1]Yayoi Kinoshita DDS, [1]Michael Donovan MD, [1,2]Yevgeniy Antipin PhD, [5]Yan Li PhD, [5]Xiaoxiao Liu MS, [5]Fulai Jin PhD, [1]Peng Wang PhD, [1,2]Andrew Uzilov PhD, [1]Carmen Argmann PhD, [1,2]Eric E. Schadt PhD, [1,±,*]Andrew F. Stewart MD, [1,±]Donald K Scott PhD,[1, ¶]Luca Lambertini PhD.

From the [1]Diabetes Obesity and Metabolism Institute, The Department of Surgery, The Department of Pathology, The Department of Genetics and Genomics Sciences and The Institute for Genomics and Multiscale Biology, The Icahn School of Medicine at Mount Sinai, New York, NY, 10029; [2]Sema4, Stamford CT 06902; [3]The Academic Endocrine Unit, University of Oxford, UK OX3 7LJ; [4]The Cancer Institute of New Jersey, New Brunswick NJ 08901, and [5]The Department of Genetics and Genome Sciences, Case Western Reserve University, Cleveland OH, 44106.

Supplementary Figures.

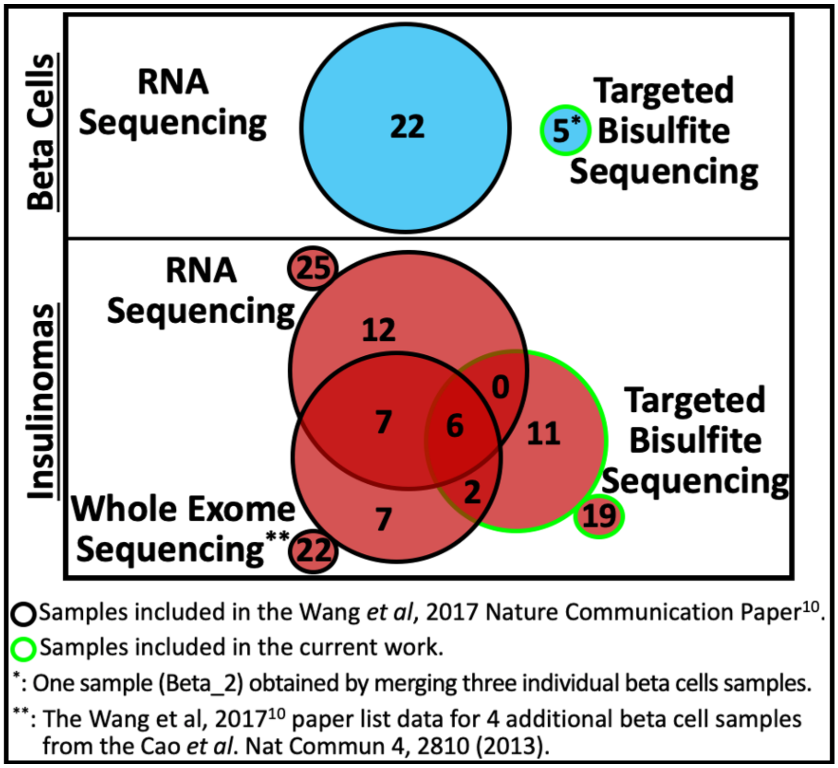

Supplementary Figure 1. Venn Diagram Illustrating the Source of Beta Cells and Insulinomas and Sequencing Approaches Employed.

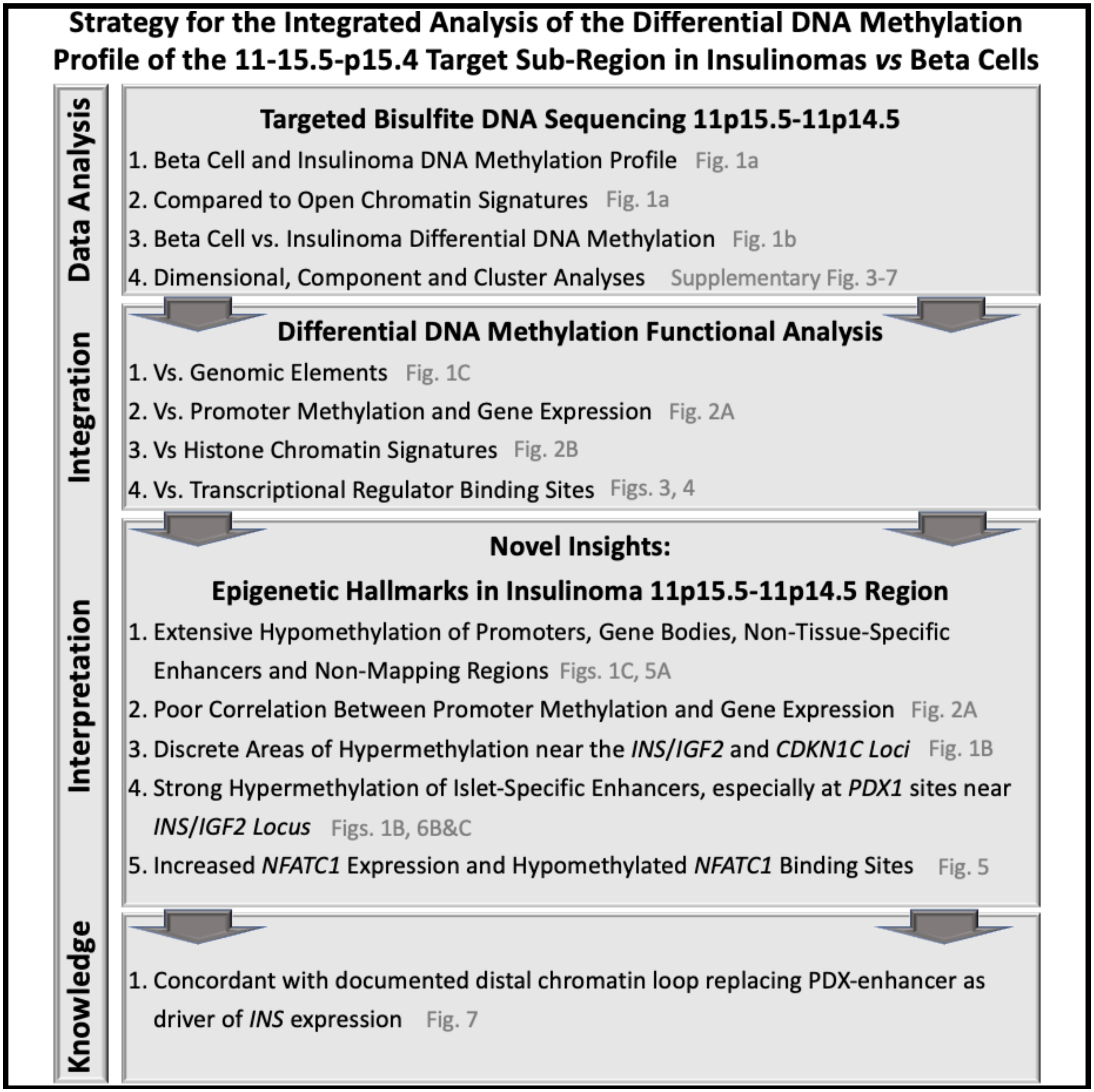

Supplementary Figure 2. Strategy for the Integrated Analysis of the DNA Methylation Profile of the 11p15.5-p15.4 Sub-Region in Insulinomas. See text for details.

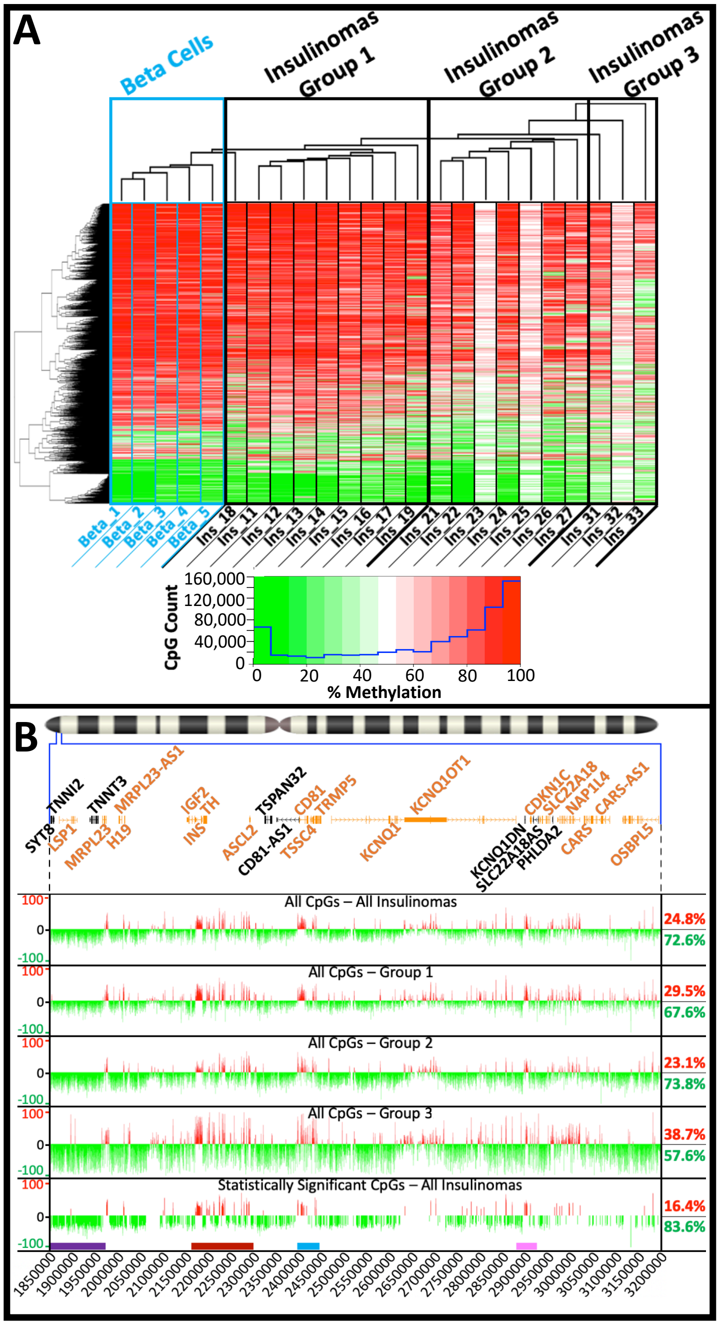

Supplementary Figure 3. Cluster Analysis Exploring Beta Cell and Insulinoma Clusters. A. Heatmap of the DNA methylation percentage for all 29,675 CpG dinucleotides that were retained after filtering and normalization (rows) and the 24 samples (columns) included in the study. Clustering trees (hclust) are shown on the top and the left of the heatmap for samples and CpG dinucleotides, respectively. Individual sample names are reported at the bottom of the heatmap in light blue (beta cells) and black (insulinomas). The dataset was principally unidimensional (see **Supplementary Figs. 4**,**5** for details), and did not identify discreet clusters of beta cells or insulinomas. **B.** As detailed in Results, insulinomas in panel A were visually sorted into three groups, 1, 2 and 3, and their three methylation tracks were generated using all DNA methylation values from all measured CpG dinucleotides (the top track in **Fig. 1B**) and statistically significant differentially methylated CpG dinucleotides (bottom track in **Fig. 1B**). Despite the visual differences among groups 1,2 and 3 in panel A, they displayed very consistent differential methylation patterns vs. beta cells.

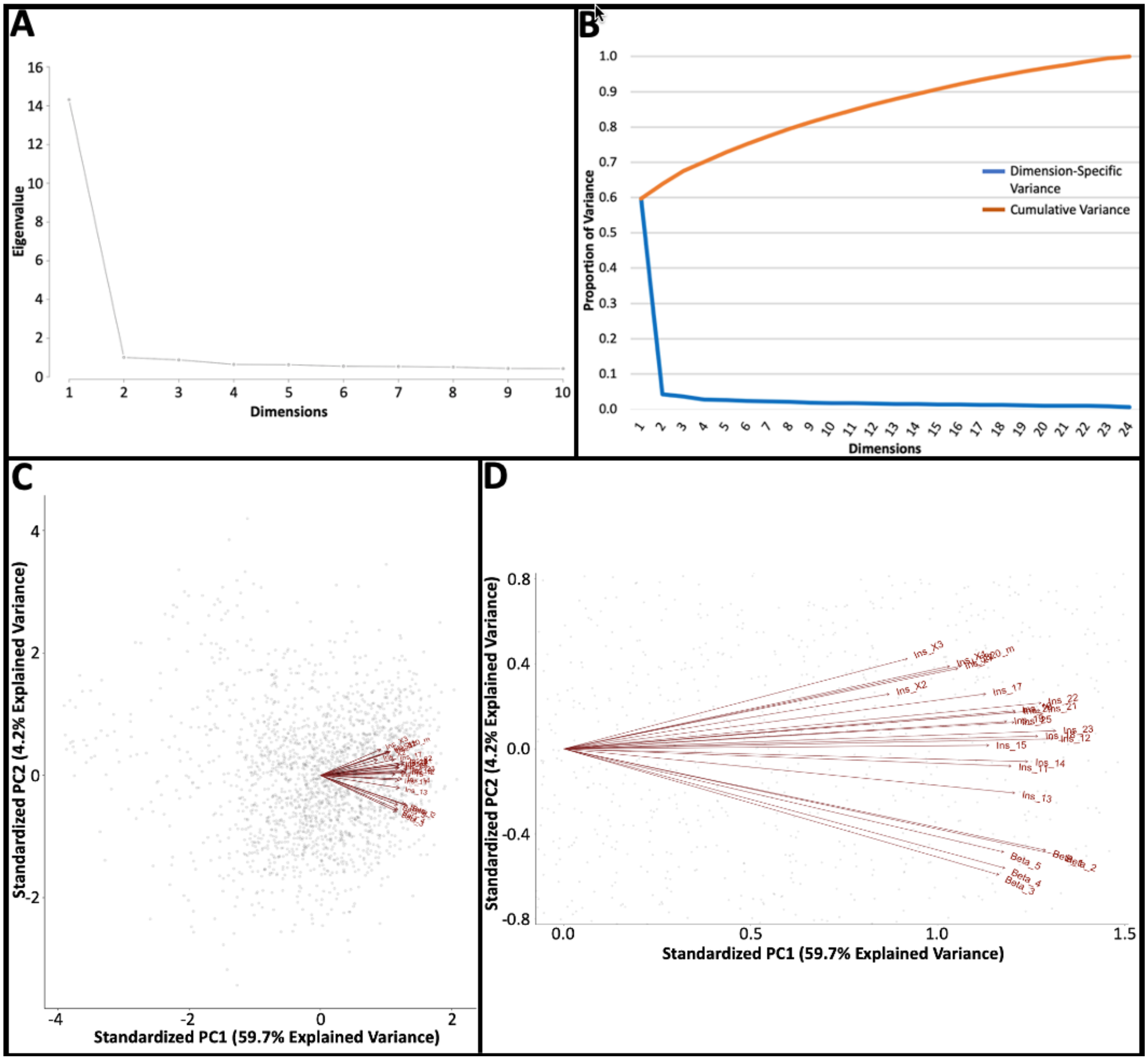

Supplementary Figure 4. DNA Methylation Dimensional Analysis. **A**. Scree plot for the dimensional analysis of all DNA methylation data. The X-axis reports the maximum (10) number of dimensions considered; the Y-axis reports the eigenvalue associated with each dimension. The data distribution of the sample clearly follows a one-dimensional structure, since the "scree" is located after the first dimension. Additional dimensions do not add significant contributions to the eigenvalue calculation. **B**. Variance plot for the dimensional analysis of all DNA methylation data. The X-axis reports all (24) dimensions considered by this analysis; the Y-axis reports the percent variance associated with each dimension. The dark red line represents the cumulative variance, and the blue line the individual variance. Again, the profile of the dataset is one-dimensional, with the first dimension accounting for approximately 60% of the total variance. **C**. PCA plot for the DNA methylation data. The X- and Y-axes report the component loadings for components 1 and 2, respectively. As expected from a one-dimensional dataset, all samples merge towards one direction, rather than highlighting specific sample clusters. **D**. Magnification of the PCA plot for the DNA methylation data. The X- and Y-axes report the component loadings for components 1 and 2, respectively, in the region of the graph carrying the component vectors for each sample. Along component 2 of the graph, which explains only about 4.1% of the total variance, two groups can be identified that separately include all beta cells (toward the bottom of the graph) and all insulinomas (in the mid and upper portion of the graph).

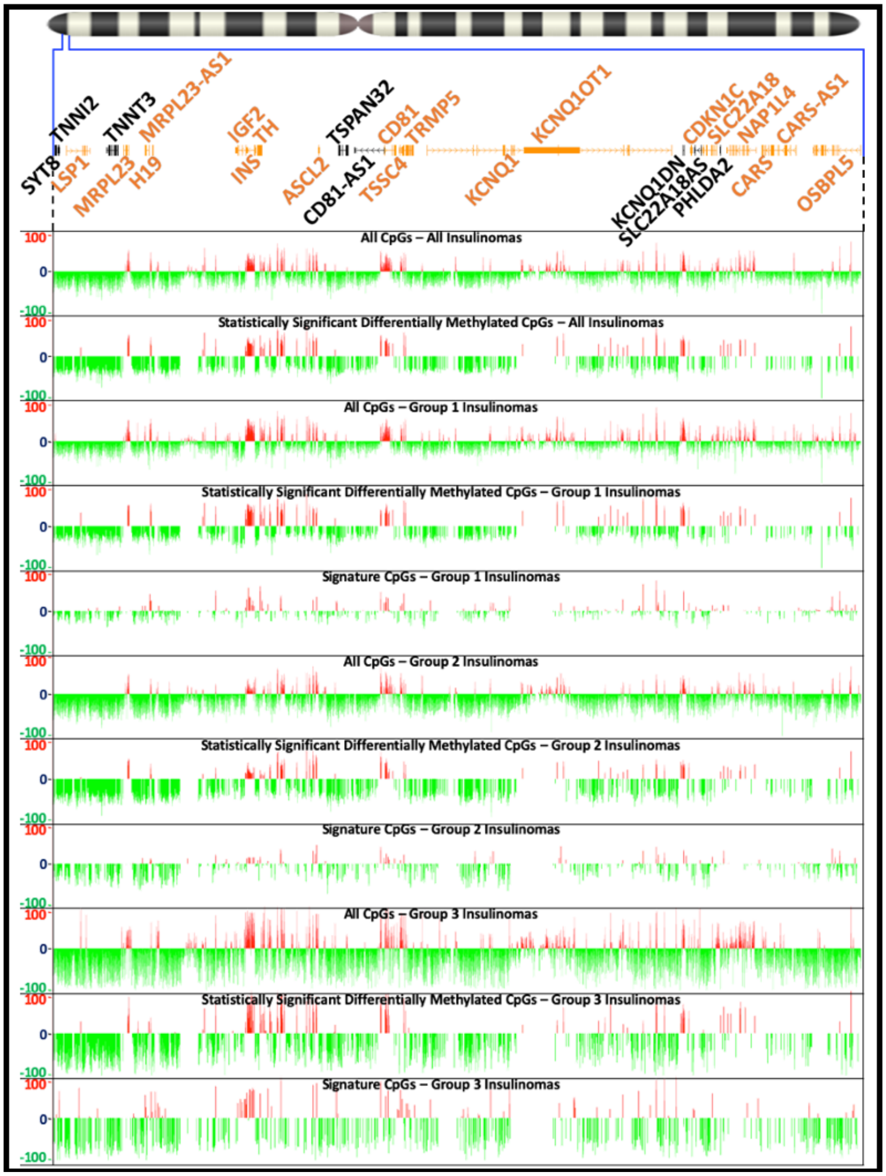

Supplementary Figure 5. **Signature Analysis for the Insulinoma Groups**. The chromosome 11 ideogram with magnification of the 11p15.5-p15.4 target sub-region is displayed at the top as in **Fig. 1A**. Following are, from top to bottom: the differential methylation tracks for all CpG dinucleotides measured and for the statistically significant CpG dinucleotides for all insulinomas (same as **Fig. 2A** top and bottom tracks respectively); per each group the differential methylation tracks for all CpG dinucleotides measured, for the statistically significant CpG dinucleotides and for the “signature” CpG dinucleotides. Signature CpG dinucleotides were obtained by: 1) discarding those CpG dinucleotides that, within each group, had a standard deviation of the differential methylation above the 95th ‰ for its distribution; 2) among the remaining CpGs dinucleotides, selecting those that are common to the three groups; 3) for each of the remaining CpG dinucleotides, calculating the standard deviation of the average differential methylation of each group; and, finally 4) selecting the bottom 95th ‰ of CpG dinucleotides with the highest standard deviation (see Methods and **Supplementary Data 3 and 5**). As expected from the results of the dimensional and component analyses, the group signatures appear quite similar.

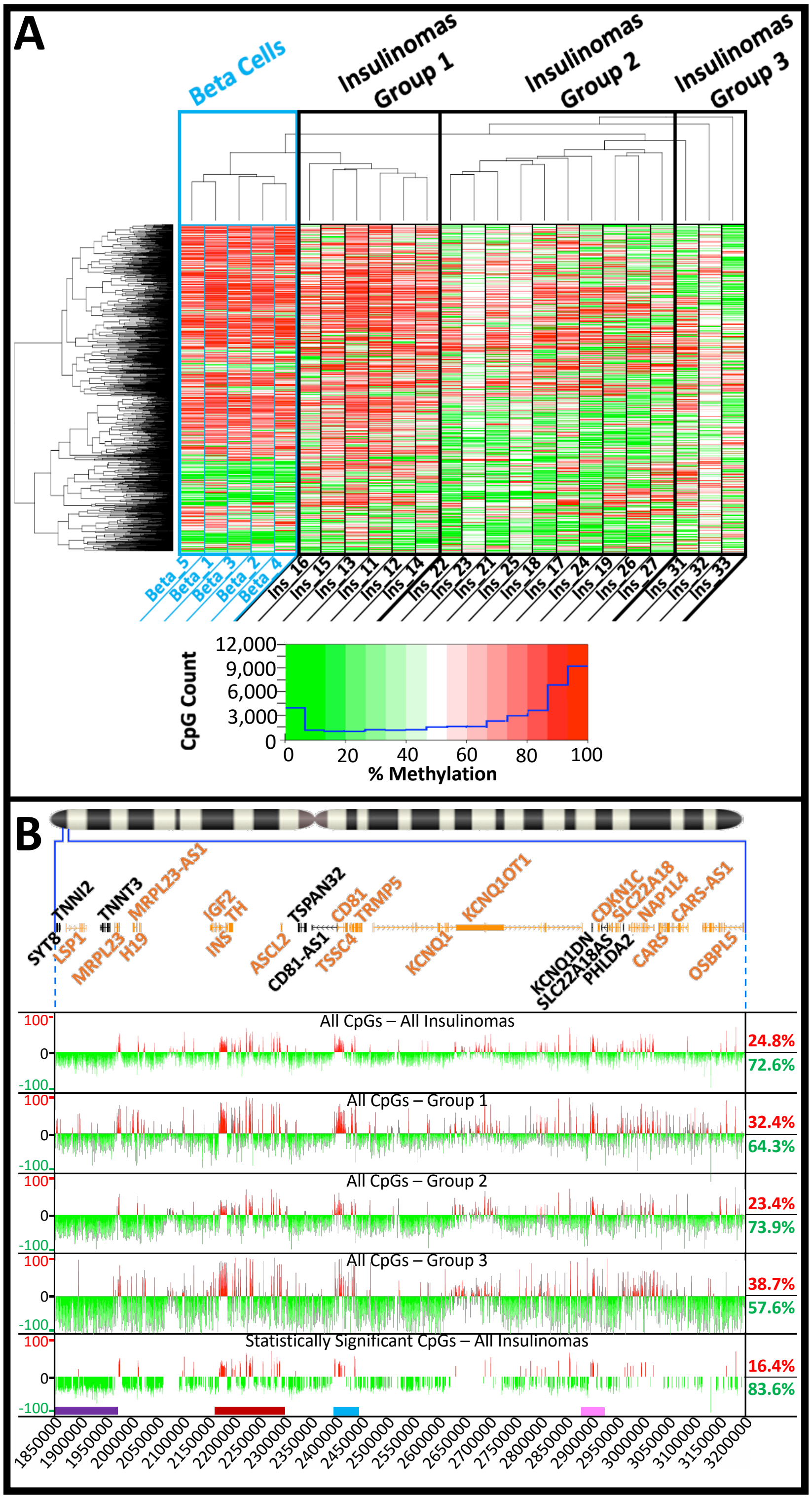

Supplementary Figure 6. Cluster Analysis of Beta Cell and Insulinoma Using the Top Variable ("TV") CpG Dinucleotides. A. Heatmap of the DNA methylation percentage for 2,850 CpG dinucleotides that show standard deviation above the 90th percentile for its distribution across all 29,675 measured CpG dinucleotides, derived using the same hclust algorithm in **Supplementary Fig. 3**. In this analysis groups, are referred to as "TV" to distinguish these groups generated by using the top variable CpGs from those in **Supplementary Fig. 3**. **B.** Differential methylation tracks generated using the "TV" groups. The tracks for the "TV" groups are very similar to those of **Supplementary Fig. 3**.

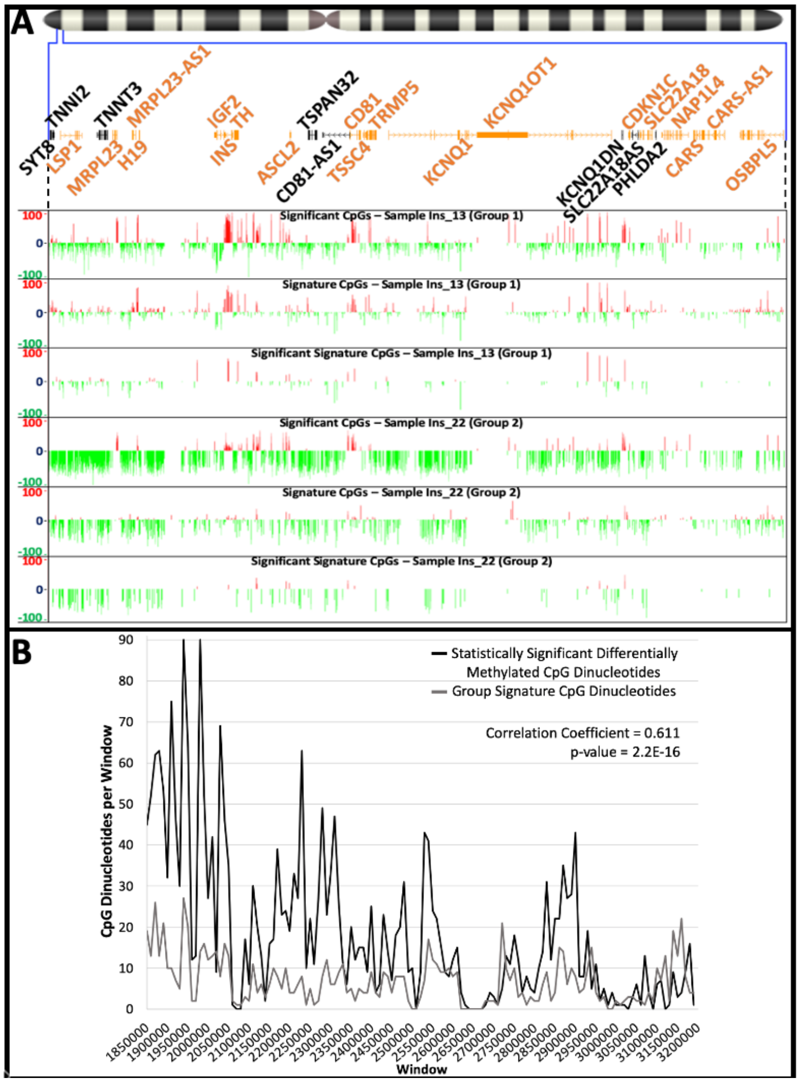

Supplementary Figure 7. **Insulinoma Groups Signatures Have a Similar Distribution to Statistically Significant Differentially Methylated CpG Dinucleotides**. **A.** Examples of the different profiles of differential methylation for significant, signature and significant signature CpG dinucleotides. The chromosome 11 ideogram with magnification of the 11p15.5-p15.4 target sub-region is displayed at the top as in **Fig. 1A**. The six tracks, for two samples, Ins_13 (Insulinoma Group 1) and Ins_22 (Insulinoma Group 2), show, from top to bottom, the differential methylation profile for the CpG dinucleotides belonging, respectively, to the significant and signature sets and for the significant CpG dinucleotides which are also part of the signature set. The two samples have been chosen because they show, within the two Insulinoma Groups, the most “extreme” profiles. It is noticeable how the signature CpG dinucleotides show, for sample Ins_13 a dominant hypermethylation, the opposite for sample Ins_22. This status appears to replicate for the significant CpG dinucleotides that are also part of the signature set underscoring a role that may be more relevant for these CpG dinucleotides in determining differences in the insulinoma phenotype (see **Supplementary Data 3**). **B.** Comparison of the distributions of the statistically significant CpG dinucleotides and the signature CpG dinucleotides. The X-axis represents a breakdown of the 1.35 Mbp target sub-region into 135 windows of 10 Kbp each. The Y-axis reports the count of CpGs per window. A noticeable degree of co-linearity can be seen between the distribution of statistically significant CpG dinucleotides (black line) and signature CpG dinucleotides (gray line) (see **Supplementary Data 5**). This may point to an overlapping between the role of statistically significant CpG dinucleotides on determining the insulinoma transformation of beta cells and the role of signature CpG dinucleotides in determining the insulinoma sub-phenotype.

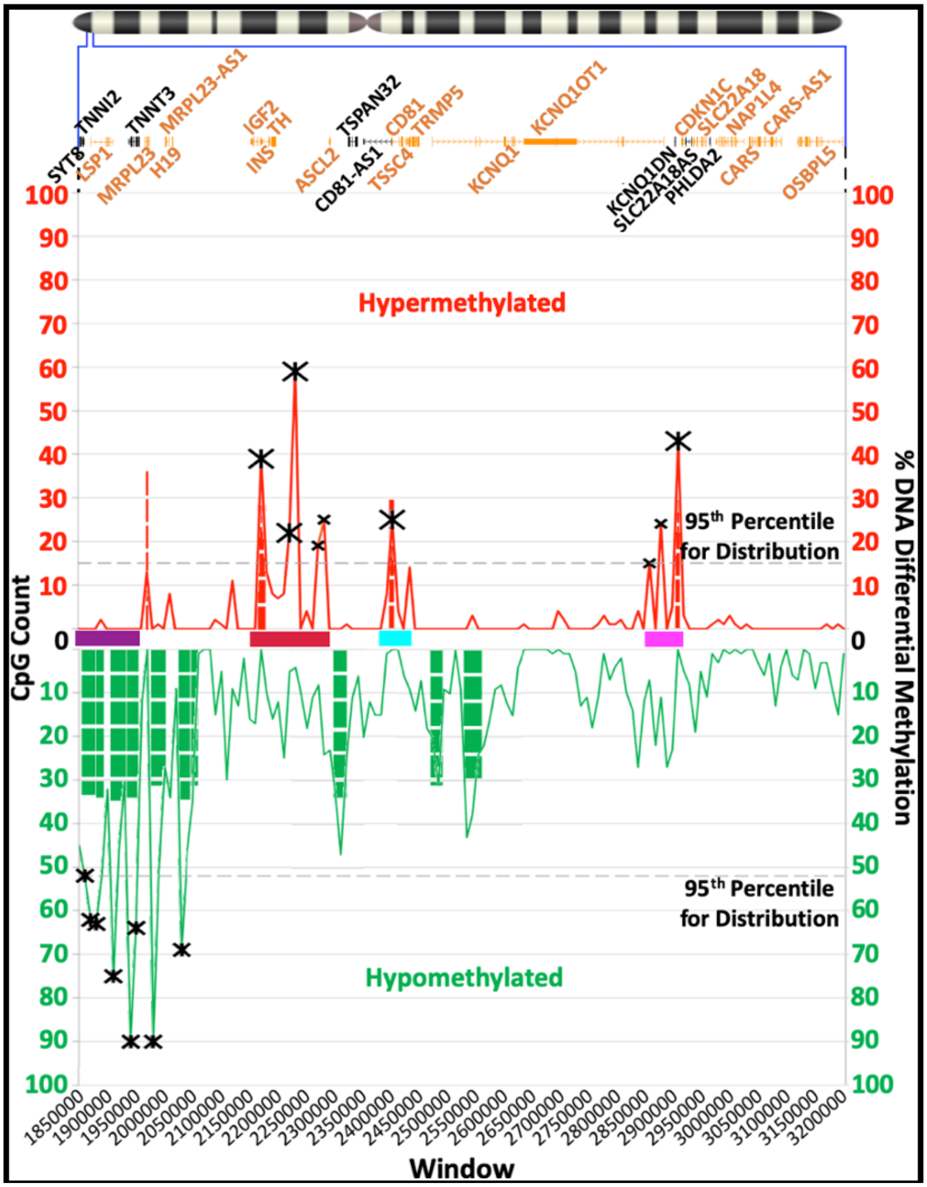

Supplementary Figure 8. **Definitions of the Regions with the Strongest Differential Methylation Profiles.** The chromosome 11 ideogram with magnification of the 1.35 Mbp 11p15.5-p15.4 target sub-region is displayed at the top as in **Fig. 1A**. The X-axis represents, for the red and green lines, a breakdown of the 1.35 Mbp target sub-region into 135 windows of 10 Kbp each. For the dashed red and green vertical bars, it refers to the specific coordinates of the differentially methylated regions (DMRs – FDR <0.005) as identified by DMRcate. The left Y-axis reports the count of CpGs per 10 Kbp window. Above and below the "0" black line the number of, respectively, hypo- and hyper-methylated CpG dinucleotides is reported as peaks. The right Y-axis reports the percent differential methylation referring to the DMRs. The purple, dark red, light blue and pink regions are highlighted in the middle of the graph, between the hypo- and hypermethylated CpG/DMR graphs. The 95th ‰ distribution for hypo- and hypermethylated CpGs dinucleotides across windows is marked with dashed gray lines. Peaks of hypo- and hypermethylated CpG dinucleotide counts that include more than 75% of the CpGs for a specific window are marked with the "✕" symbol, while peaks that also cross the 95th ‰ distribution of hypo- and hyper-methylated CpGs across windows are marked with the "⁎" symbol. The DMR and CpG dinucleotide distributions and the hypo-/hypermethylation peaks ultimately define the coordinates of purple, dark red, light blue and pink regions that we separately surveyed (see **Supplementary Data 5 and 6**).

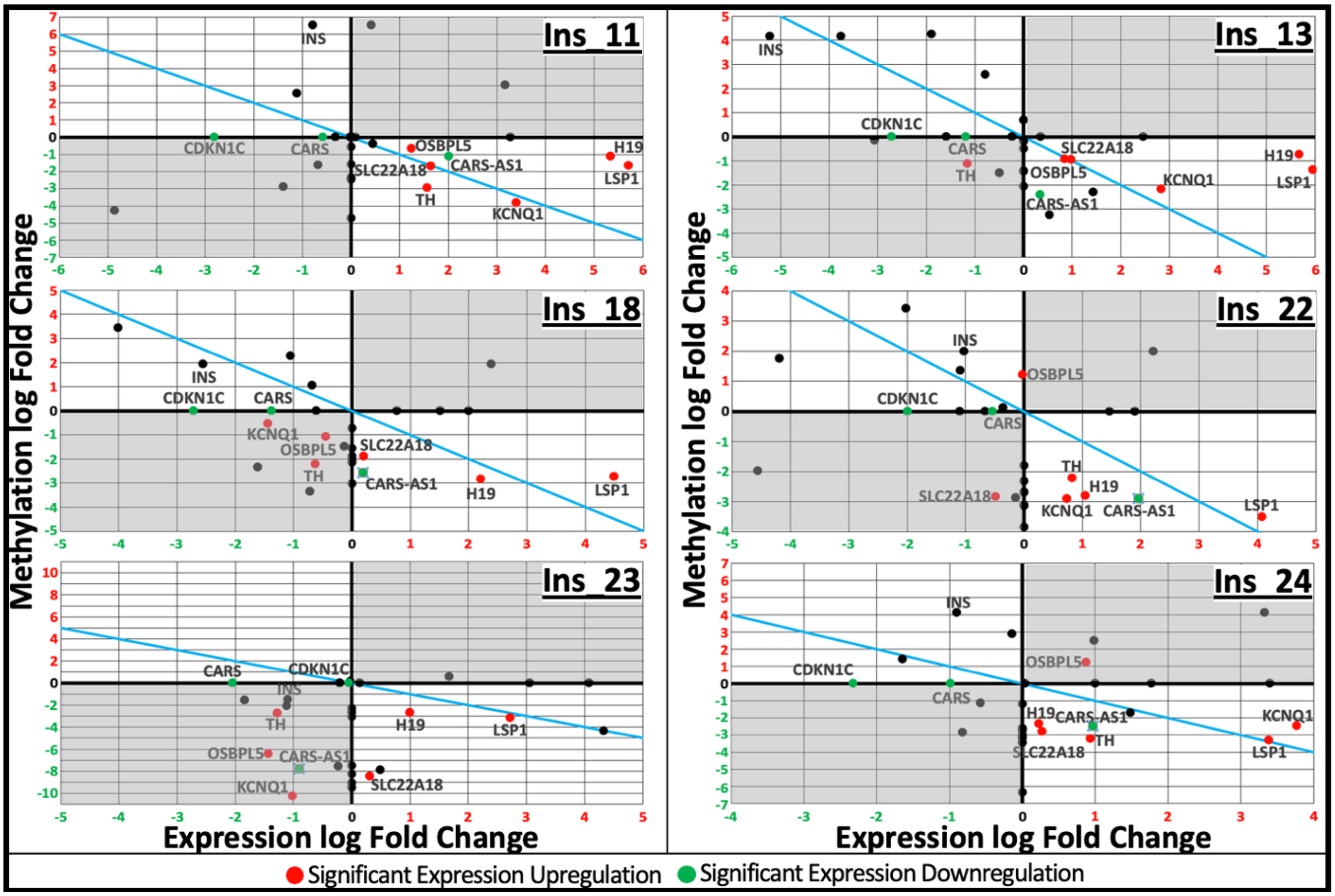

Supplementary Figure 9. Sample-Specific Promoter DNA Methylation Versus Gene Expression for the Six Insulinomas with both RNA-seq and Bisulfite DNA sequencing data. This supplementary figure supports the findings shown in **Fig. 2**. Even samples with both gene expression and promoter DNA methylation data show poor correlation between the two measurements. The six charts of this panel are built as for **Fig. 2A** with the Y-axis reporting the DNA methylation log-fold change and the X-axis the expression log-fold change. DNA methylation and gene expression changes have been obtained by comparing the DNA methylation and gene expression values for each insulinoma to the average of all beta cells of each respective analysis. For each sample, the number of genes with concordant expression and promoter DNA methylation is similar to the average across all samples. Note that genes are, as for their expression change, labeled accordingly to **Fig. 2A** and our published work[1]. A full comparison of the consistency between promoter DNA methylation and gene expression is reported in **Supplementary Data 10** that further underscores how the DNA methylation status is constant across samples, even more than the gene expression. Overall in 4 out of 6 samples methylation and expression show the same direction as in the average of **Fig. 2A**. Note that because of the sample-specific approach, these data show an elevated level of variability resulting from the inability to use the appropriate correctional statistical tools used in processing sample sets.

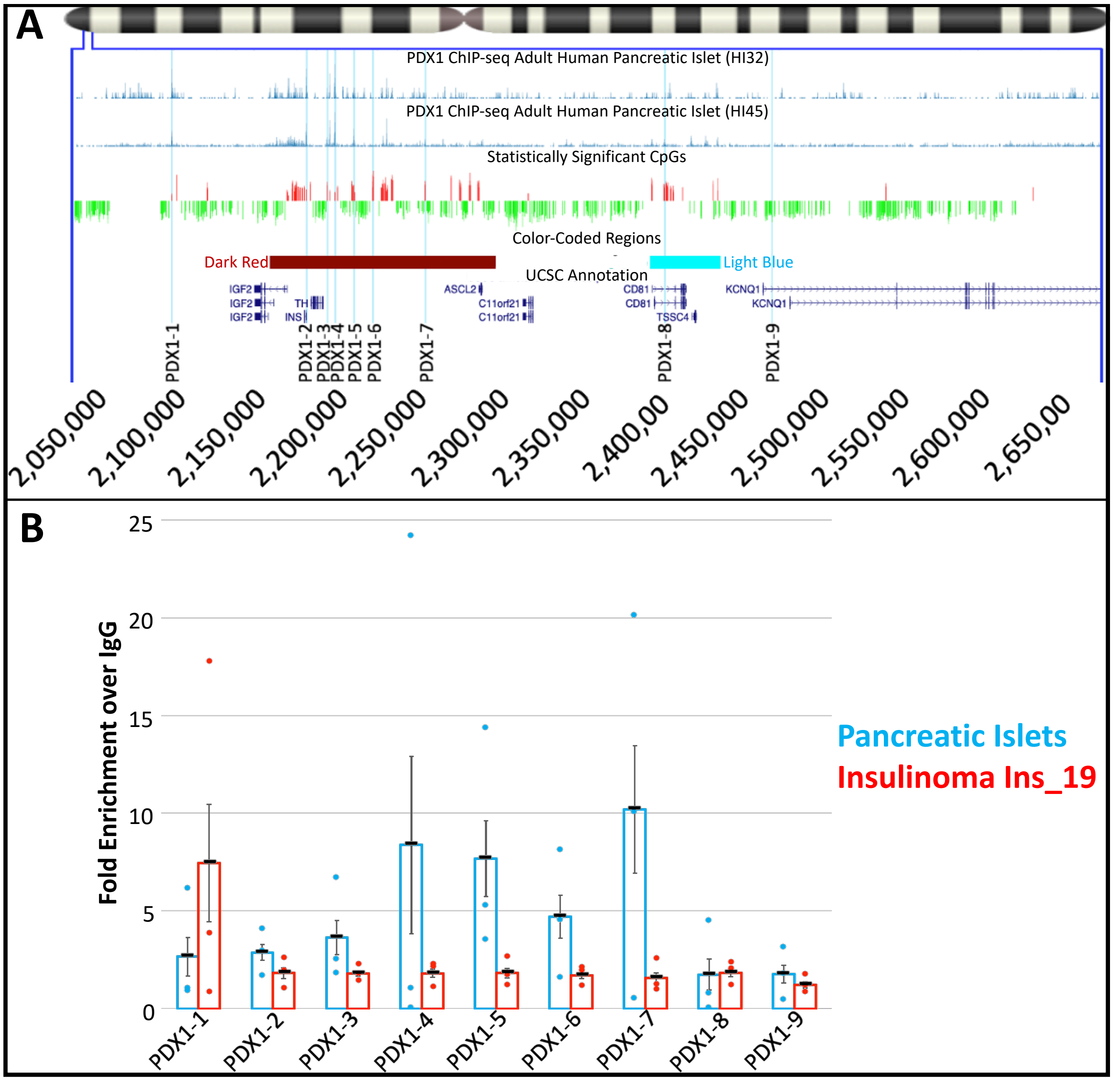

Supplementary Figure 10. ChIP-qPCR Analysis for *PDX1* Binding in Human Pancreatic Islets and Insulinoma. ChIP-qPCR analysis of *PDX1* binding sites in dispersed pancreatic human islets from three different donors vs. insulinoma Ins_19 (see **Supplementary Data 14** for primer information). **A**. Genome browser view of the region including the location of the 9 *PDX1* sites chosen for the analysis highlighted with blue vertical lines. Sites 1 to 8 are taken from the hypermethylated set of binding sites reported in **Figure 5B** (top row) while site 9 (negative control) comes from the set of non-differentially methylated binding sites of the same figure. The two top tracks show *PDX1* ChIP-seq data from human pancreatic islets by Pasquali *et al*[2]. The third track illustrates the statistically significant differentially methylated CpG dinucleotides of the region shown here and they are derived from **Fig. 2A** (bottom track). Color-coded hypermethylated dark red and light blue region footprints are shown right below while the bottom track shows the UCSC gene annotations. *PDX1* sites are labeled and coordinates provided at the bottom of the panel. **B**. Histogram/dot plot for the ChIP-qPCR analysis of PDX1 binding sites of the three different human islet samples vs. insulinoma Ins_19. In human pancreatic islets binding of *PDX1* occurs at known *PDX1* binding sites as expected (blue bars and dots). In contrast, *PDX1* binding to the same regions in a human insulinoma is undetectable for sites 2 to 7 (red bars and dots), which are all within the hypermethylated dark red region. Error bars indicate mean ± SEM.

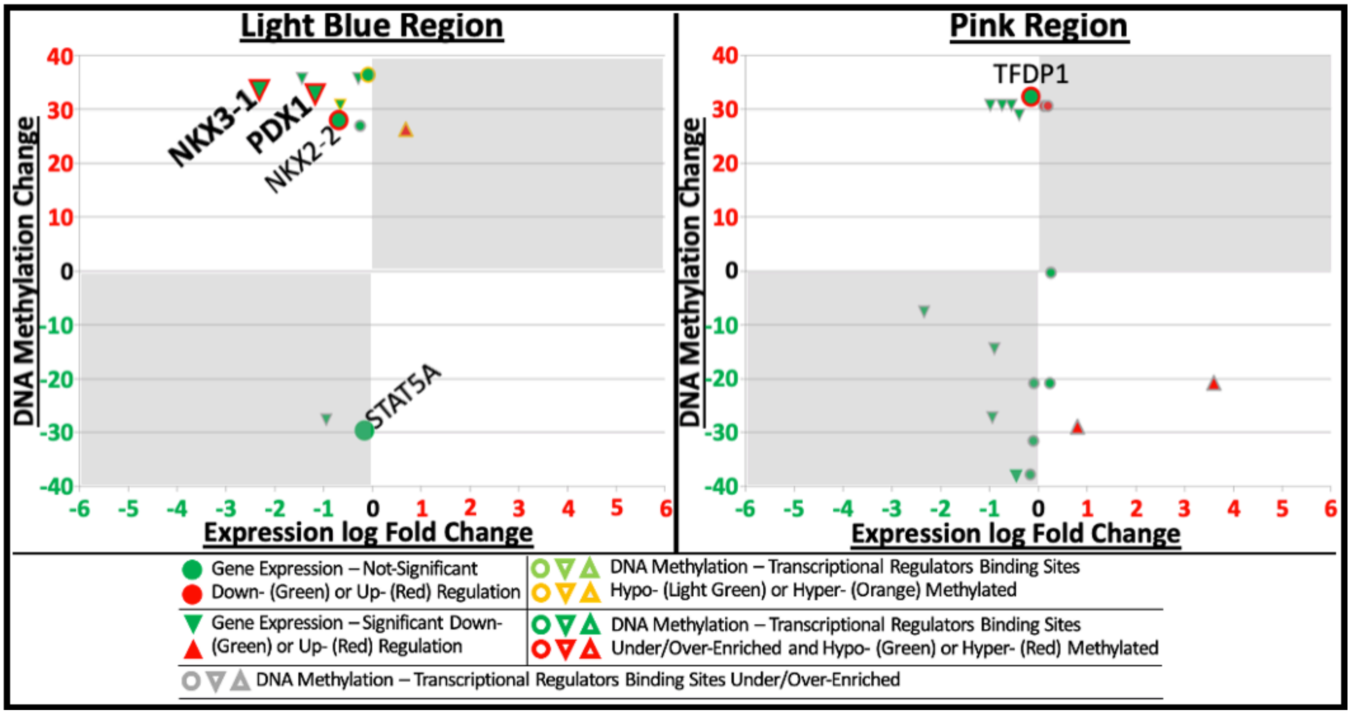

Supplementary Figure 11. **Key Transcriptional Regulator Binding Sites for the Light Blue and Pink Regions.** This graph was created identically to that displaying the purple and dark red regions in **Fig. 6A**. As observed for the dark red region in **Fig. 6A**, in the light blue region *PDX1* and *NKX3-1* also shows both a significant reduction in expression, as well as strong cumulative hypermethylation of their cognate binding sites. Note also that the light blue region, as compared to the dark red and purple regions, contains substantially fewer transcriptional regulator binding sites displaying differentially methylated CpG dinucleotides. The pink region displays a completely different profile that does not highlight any transcriptional regulator binding sites showing strong differential enrichment/methylation (<5th ‰ and >95th ‰) accompanied by concordant and significant changes in gene expression (see **Supplementary Data 15 and 17**).

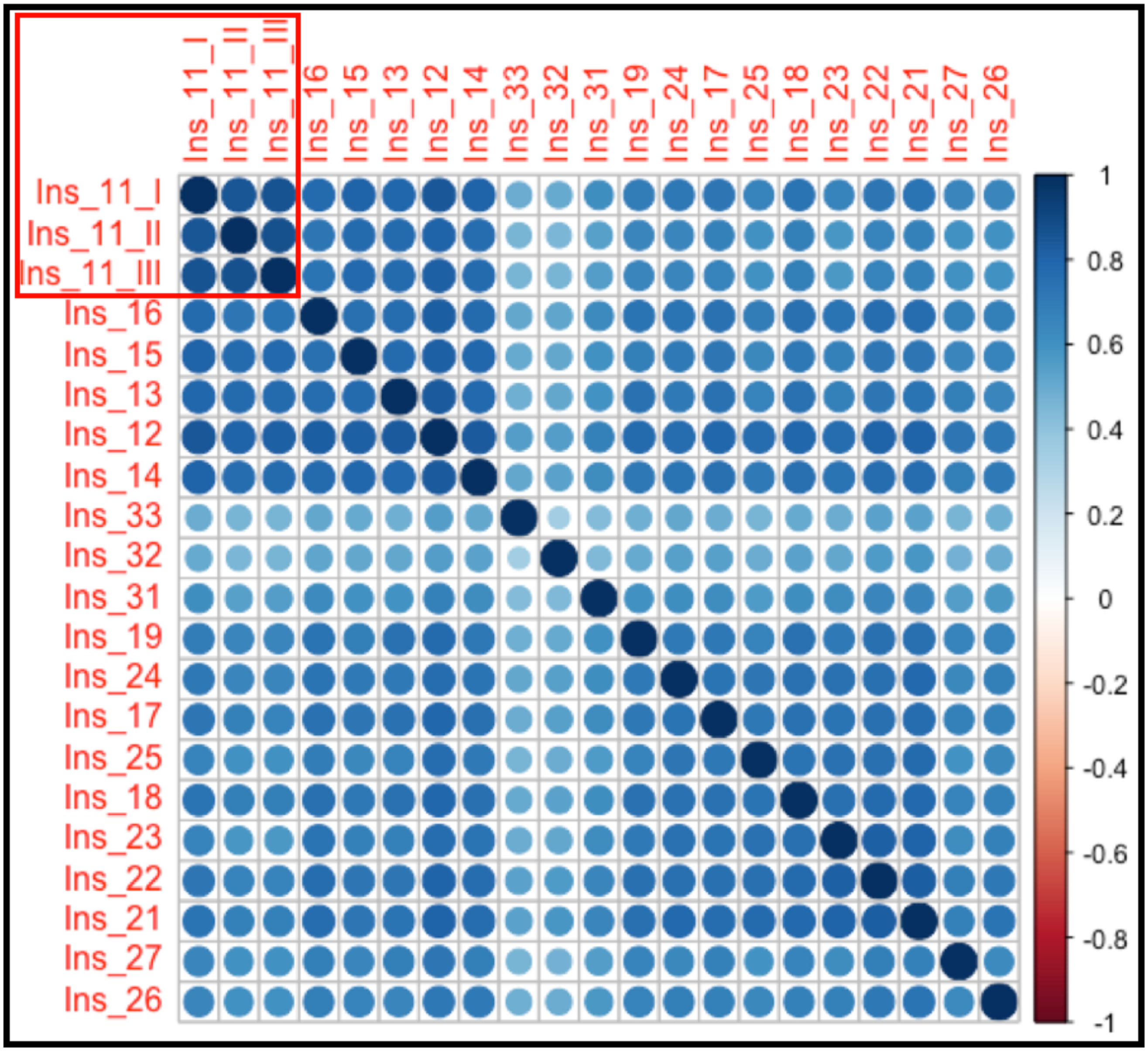

Supplementary Figure 12. **Consistency of Sequencing Fidelity Across Runs.** The matrix represents the correlation between the methylation profiles across all measured CpG dinucleotides for all the insulinomas analyzed and the three replicates of sample Ins_11. Sample Ins_11 was analyzed in each of the three multiplexed sequencing runs (Ins_11_I, Ins_11_II and Ins_11_III in this matrix). When ranking the correlation matrix using the hclust algorithm, the three replicates of sample Ins_11 cluster tightly together showing a correlation that is higher than those with any of the other insulinomas.

Supplementary References

1. Wang, H. *et al.* Insights into beta cell regeneration for diabetes via integration of molecular landscapes in human insulinomas. *Nature communications* **8**, 767, doi:10.1038/s41467-017-00992-9 (2017).
2. Pasquali, L. *et al.* Pancreatic islet enhancer clusters enriched in type 2 diabetes risk-associated variants. *Nature genetics* **46**, 136-143, doi:10.1038/ng.2870 (2014).
