# Supplementary material for: Aberrant methylation underlies insulin gene expression in human insulinoma
Source: Nat Commun. 2020 Oct 15;11:5210. doi: 10.1038/s41467-020-18839-1 (PMC7566641; doi:10.1038/s41467-020-18839-1)
Supplement: Supplementary file 3 — Description of Additional Supplementary Files [file 41467_2020_18839_MOESM3_ESM.pdf]

File Name: Supplementary Data 1
Description: **Normal Beta Cell Identifiers and Demograpics.**

File Name: Supplementary Data 2
Description: **Insulinoma Identifiers and Clinical Features.**

File Name: Supplementary Data 3
Description: **Summary Table for All CpG Dinucleotides Measured Across all Samples Including: Percent DNA Methylation Per Sample; Percent Average Methylation for Beta Cells, all Insulinomas and each Insulinoma Group; Differential Methylation Insulinomas Vs Beta Cells for all Insulinomas (with p and FDR values) and each Insulinoma Group; Labeling of Statistically Significant and Signature CpG Dinucleotides.**

File Name: Supplementary Data 4
Description: **Average DNA Methylation and Average Maximum ATACseq Peaks for the 135 10 kbp-windows of the 11p15.5-p15.4 target sub-region.**

File Name: Supplementary Data 5
Description: **Distribution of the Statistically Significant and Signature CpG Dinucleotides by the 135 10 kbp-windows of the 11p15.5-p15.4 target sub-region.**

File Name: Supplementary Data 6
Description: **Characteristics of all Differentially Methylated Regions Scored by DMRcate.**

File Name: Supplementary Data 7
Description: **List of All Genomic Elements Cosidered by this Study.**

File Name: Supplementary Data 8
Description: **Annotation of the Statistically Significant CpG Dinucleotides by Genomic Element.**

File Name: Supplementary Data 9
Description: **Methylation Index, Methylation log-Fold Change and Expression log-Fold Change for all Gene Isoforms of the 11p15.5-p15.4 target sub-region with either DNA Methylation or Gene Expression Data Available.**

File Name: Supplementary Data 10
Description: **Comparison between promoter DNA methylation and gene expression for the six samples with both RNA-seq and bisulfite DNA seq. For promoters of genes which are not expressed, the average promoter DNA methylation across all isoforms has been considered. For all other genes the promoter isoform with the best possible correlation with gene expression has been considered.**

File Name: Supplementary Data 11
Description: **Promoter, Enhancer and Dyadic Chromatin Signatures Coverage from Roadmap Epigenomics for the 11p15.5-p15.4 Target Sub-Region over 10 Kbp Windows.**

File Name: Supplementary Data 12
Description: **Cumulative Differential Methylation and Transcriptional Regulator Binding Site Enrichment Scores for All and for the Statistically Significant Differentially Methylated CpG Dinucleotides of the 11p15.5-p15.4 traget sub-region.**

File Name: Supplementary Data 13
Description: **List of all Transcriptional Regulator Binding Sites with Strong Signatures of Hypo-/Hypermethylation and Under/Over-Enrichment.**

File Name: Supplementary Data 14
Description: **Primer sets for the ChIP-qPCR testing on 9 PDX1 binding sites across the 11p15.5-p15.4 target sub-region.**

File Name: Supplementary Data 15
Description: **Transcriptional Regulator Binding Sites Average Differential Methylation Vs Gene Expression.**

File Name: Supplementary Data 16
Description: **Expression values for PDX1, NKX3-1 and NFATC1 in beta cells and insulinomas. From Wang et al, 2017 (ref #10)**

File Name: Supplementary Data 17
Description: **List of All Sites for Transciptional Regulators PDX1, NKX3-1, NFATC1 and Differential Methylation Status.**

File Name: Supplementary Data 18
Description: **Annotation of All Transcriptional Regulator Binding Sites Carrying Statistically Significant Differentially Methylated CpG Dinucleotides by Genomic Element.**

File Name: Supplementary Data 19
Description: **Distribution of the Transcriptional Regulator Binding Sites and of the Scores from the 4C-Seq Study on EndoC-βH1 Cells across the 135 10 kbp-windows of the 11p15.5-p15.4 target sub-region.**
