# Supplementary material for: Aberrant methylation underlies insulin gene expression in human insulinoma
Source: Nat Commun. 2020 Oct 15;11:5210. doi: 10.1038/s41467-020-18839-1 (PMC7566641; doi:10.1038/s41467-020-18839-1)
Supplement: Supplementary file 23 — Reporting Summary [file 41467_2020_18839_MOESM23_ESM.pdf]

nature research

Corresponding author(s): Andrew F Stewart

Last updated by author(s): 2020/08/28

Reporting Summary

Nature Research wishes to improve the reproducibility of the work that we publish. This form provides structure for consistency and transparency in reporting. For further information on Nature Research policies, see our Editorial Policies and the Editorial Policy Checklist.

Statistics

For all statistical analyses, confirm that the following items are present in the figure legend, table legend, main text, or Methods section.

n/a	Confirmed	
☐	☒	The exact sample size (*n*) for each experimental group/condition, given as a discrete number and unit of measurement
☐	☒	A statement on whether measurements were taken from distinct samples or whether the same sample was measured repeatedly
☐	☒	The statistical test(s) used AND whether they are one- or two-sided<br>*Only common tests should be described solely by name; describe more complex techniques in the Methods section.*
☒	☐	A description of all covariates tested
☒	☐	A description of any assumptions or corrections, such as tests of normality and adjustment for multiple comparisons
☐	☒	A full description of the statistical parameters including central tendency (e.g. means) or other basic estimates (e.g. regression coefficient) AND variation (e.g. standard deviation) or associated estimates of uncertainty (e.g. confidence intervals)
☐	☒	For null hypothesis testing, the test statistic (e.g. *F*, *t*, *r*) with confidence intervals, effect sizes, degrees of freedom and *P* value noted<br>*Give P values as exact values whenever suitable.*
☒	☐	For Bayesian analysis, information on the choice of priors and Markov chain Monte Carlo settings
☐	☒	For hierarchical and complex designs, identification of the appropriate level for tests and full reporting of outcomes
☐	☒	Estimates of effect sizes (e.g. Cohen's *d*, Pearson's *r*), indicating how they were calculated

Our web collection on statistics for biologists contains articles on many of the points above.

Software and code

Policy information about availability of computer code

Data collection	R version 3.5.3 - CASAVA version 1.8.2 - BSMAP version 2.89 - bedtools version 2.29.1
Data analysis	R version 3.5.3 - Packages: corrplot - version 0.84 - gplots version 3.0.4 - DMRcate version 2.2.1 - bedr version 1.0.7

For manuscripts utilizing custom algorithms or software that are central to the research but not yet described in published literature, software must be made available to editors and reviewers. We strongly encourage code deposition in a community repository (e.g. GitHub). See the Nature Research guidelines for submitting code & software for further information.

Data

Policy information about availability of data

All manuscripts must include a data availability statement. This statement should provide the following information, where applicable:

- Accession codes, unique identifiers, or web links for publicly available datasets
- A list of figures that have associated raw data
- A description of any restrictions on data availability

The methylome DNA sequencing data have been deposited in the NIH/NIDDK Diabetes Genotype and Phenotype (dbGaP) database [https://www.ncbi.nlm.nih.gov/projects/gap/cgi-bin/about.html] under the accession code phs001422.v1.p1 [https://www.ncbi.nlm.nih.gov/gap/advanced_search/?TERM=phs001422.v1.p1]. The source data underlying Figure 1, Supplementary Figures 3, 5, 6 and 7 and Supplementary Data 3 are provided in dbGaP. All the other data supporting the findings of this study are available within the article and its supplementary information files and from the corresponding author upon reasonable request. A reporting summary for this article is available as a Supplementary Information file.

Field-specific reporting

Please select the one below that is the best fit for your research. If you are not sure, read the appropriate sections before making your selection.

- [x] Life sciences
- [ ] Behavioural & social sciences
- [ ] Ecological, evolutionary & environmental sciences

For a reference copy of the document with all sections, see nature.com/documents/nr-reporting-summary-flat.pdf

Life sciences study design

All studies must disclose on these points even when the disclosure is negative.

Sample size	No sample size calculation was conducted because of the limited number of sorted beta cells and insulinomas available. We applied stringent statistical criteria for selecting significant differentially methylated CpGs (FDR < 0.005) to compensate for the limited numbr of samples available. We also conducted clustering, component and dimensional analyses to verify the integrity and reliability of our data.
Data exclusions	The only exclusion criterion applied was that of selecting CpGs with reading depth equal or greater than 5X in order to guarantee for reliable CpG methylation calls. This criterion is routinely applied and therefore determined in advance of any data analysis while processing raw data.
Replication	We conducted a sequencing consistency test by using one of the insulinoma samples to be run across the different sample processing, sequencing and raw data analysis. results from this technical replicate were reported in th paper (Supplementary Figure 12) and successfully describe the results of our replication approach. Results from only one, randomly chosen replicate, have been used for th rest of our analyses while comparing beta cells and insulinomas.
Randomization	The single criterion for participants' allocation was the diagnosis of insulinoma. The allocation of samples to each group was sequential following the order with which they were acquired.
Blinding	Personnel that conducted the bisulfite processing and sequencing of all samples were blinded about the group to which each sample belongs to. This guaranteed for and unbiased sample processing. For sequencing, the distribution of samples for each run was decided by th research team in order to have an equal distribution of beta cell samples and insulinomas in each multiplex run, without communicating to the personnel conducting the experimental work the group allocation of each sample.

Reporting for specific materials, systems and methods

We require information from authors about some types of materials, experimental systems and methods used in many studies. Here, indicate whether each material, system or method listed is relevant to your study. If you are not sure if a list item applies to your research, read the appropriate section before selecting a response.

Materials & experimental systems

n/a	Involved in the study	
[ ]	[x]	Antibodies
[x]	[ ]	Eukaryotic cell lines
[x]	[ ]	Palaeontology and archaeology
[x]	[ ]	Animals and other organisms
[ ]	[x]	Human research participants
[x]	[ ]	Clinical data
[x]	[ ]	Dual use research of concern

Methods

n/a	Involved in the study	
[x]	[ ]	ChIP-seq
[x]	[ ]	Flow cytometry
[x]	[ ]	MRI-based neuroimaging

Antibodies

Antibodies used	Only one antibody was used, a PDX1 antibody raised by Dr. Christopher Wright at Vanderbilt University (AB2027, BCBC - Beta Cell Biology Consortium), with a 1:200 dilution as described in Methods under "Chromatin Immunoprecipitation Assay".
Validation	This antibody has been used and validated for ChIP and ChIPseq by Pasquali et al (Pasquali L, Gaulton KJ, Rodriguez-Segui SA, Mularoni L, Miguel-Escalada I, Akerman I, Tena JJ, Moran I, Gomez-Marin C, van de Bunt M, et al. Pancreatic islet enhancer clusters enriched in type 2 diabetes risk-associated variants. Nature Genetics 46, 136-143, doi:10.1038/ng.2870 (2014) -ref 14). Pasquali et al ascertained expected cell-specific and subcellular epitopes patterns by protein blotting on human islet extracts and dual immunofluorescense of human pancreas.

Human research participants

Policy information about studies involving human research participants

Population characteristics	Sorted beta cell group<br>The mean age of the four individual beta cell donors was 41.8 +/- 5.4 (mean +/- SEM); three were male and one was female. A fifth sample was included that is the result of the pooling of three additional beta cell samples.<br>Additional clinical summary variables are presented in Supplementary Data 1 of the paper.<br>Insulinoma group<br>The mean age was 47.9 +/- 1.0 (mean +/- SEM); 12 were male and 7 were female.<br>Additional clinical summary variables are presented in Supplementary Data 2 of the paper.
Recruitment	Beta cells were isolated from seven human cadaveric islets donors provided by the NIH/NIDDK-supported Integrated Islet Distribution Program (IIDP) (https://iidp.coh.org/overview.aspx), the University of Chicago and the Alberta Diabetes Institute.<br>Nineteen insulinomas were collected from subjects who provided informed consent and were deposited in the Icahn School of Medicine at Mount Sinai Biorepository.<br>Beta cells acquisition did not imply any selection as the first samples available were acquired. The similarities between DNA methylation profiles highlights the unlikely introduction of selection bias for these samples. Similarly insulinomas were acquired from different sources as they became available using as reference the diagnoses provided by the sample source. The cluster analysis revealed high similarly between methylation profiles of the samples and the visual grouping conducted did not highlight group membership bias (see Supplementary Figures 3 to 6).
Ethics oversight	NIH/NIDDK-supported integrated Islet Distribution Program (IIDP) (https://iidp.coh.org/overview.aspx)<br>The University of Chicago<br>The Alberta Diabetes Institute<br>The Icahn School of Medicine at Mount Sinai

Note that full information on the approval of the study protocol must also be provided in the manuscript.
